# Modulation of the Immune Response by Nematode Derived Molecules

**DOI:** 10.3390/ijms26125600

**Published:** 2025-06-11

**Authors:** Michael Stear, Marta Maruszewska-Cheruiyot, Katarzyna Donskow-Łysoniewska

**Affiliations:** 1 Department of Ecological, Plant and Animal Sciences, Agribio, La Trobe University, Melbourne, VIC 3086, Australia; 2 Department of Experimental Immunotherapy, Faculty of Medicine, Lazarski University, 02-662 Warsaw, Poland; m.maruszewska@lazarski.edu.pl (M.M.-C.); k.donskow-lysoniewska@lazarski.edu.pl (K.D.-Ł.)

**Keywords:** nematode, immunomodulation, immunity

## Abstract

Parasitic nematodes produce a variety of molecules that modulate the immune system of their hosts. Over 30 molecules have been identified from more than a dozen nematode species. Some molecules are present in many species; immunomodulation has been demonstrated in some species and is assumed to exist in the remainder. Other immunomodulators appear to exist in only one or a few closely related species. The well studied nematodes produce multiple molecules to modulate the immune response and there is considerable synergy among these molecules. It is not clear why immunomodulation is so complex; possible explanations include more precise control of the host immune response or evasion of host responses against individual molecules.

## 1. Introduction

One of the most exciting recent advances in parasitology has been the identification of molecules produced by parasitic nematodes to modulate the immune response against them [1,2,3,4]. Over 30 different molecules have been identified from a variety of nematodes [1] although many more molecules are likely to exist. This research is exciting because it deepens our understanding of the host–parasite interaction and provides tools to explore the immune response in more detail. Nematodes cause disease and death in humans, domestic animals including livestock, and wildlife. They are controlled by anthelmintic treatment but this is threatened by the widespread evolution of drug resistance in parasite populations [5]. An enhanced understanding of nematode-derived immunomodulators could also lead to better parasite control.

These immunomodulatory molecules may also have a therapeutic role because aberrant immune and inflammatory responses are responsible for a variety of severe disorders including autoimmune disease, life-threatening allergic reactions and debilitating diseases such as inflammatory bowel disease. A major advantage of these molecules is their lack of toxicity, even at high concentrations. This is probably a result of the long coevolution of hosts and nematodes; selection is unlikely to favour molecules which poison their hosts.

However, controlled infection with nematodes or with some of the immunomodulators produced by nematodes has not always been effective in reducing autoimmunity [6,7]. The research on immunomodulatory molecules has attracted a number of excellent reviews; some have provided overviews [3,8,9], while some have focussed on specific cellular targets [10] and others have focussed on specific immunomodulatory molecules found in several species of nematodes such as macrophage inhibitory factors [11] or galectin [12,13].

The major focus has been, not unreasonably, on their immunomodulatory potential and less on the way the nematode uses a combination of molecules to manipulate the host immune system. However, a better understanding of the way nematodes manipulate the host could lead to more effective management of the immune system in patients with immunological and inflammatory disorders. Therefore, this review will summarise how nematode-derived molecules affect the immune system and discuss how the molecules combine to influence host immunity.

To emphasise the interactions among immunomodulators, they will be discussed nematode by nematode (Table 1). The order in which the nematodes are discussed will follow their phylogenetic relationships [14]. Several immunomodulatory molecules have been identified in more than one nematode species although activity has not been confirmed in all species. These molecules will be discussed in one place and cross-referenced to other species. Table 1 lists the nematode species which produce molecules that have been shown to be immunomodulatory and the target cell or molecule is listed. However, this research is relatively new and it is possible that these molecules may have additional targets.

## 2. Results

Clade I contains the genera Trichuris and Trichinella. The trichurid nematodes are commonly known as whipworms because the posterior part is thicker giving a visual similarity to a whip. Their products have been recently reviewed [15] and they are among the best understood of all host–nematode interactions.

The genus Trichuris contains several closely related species including *Trichuris suis*, *T. muris* and the important parasite of humans *T. trichiura.* These species have similar life cycles but different hosts. Eggs are excreted in the faeces and embryos develop into first stage larvae. Following ingestion by a host, the first stage larvae hatch, invade the mucosa of the large intestine and moult four times developing into adults over 40–45 days. *T. suis* and *T. muris* are studied as models of the human infection. Although *T. suis* is very similar to the human whipworm *T. trichiura*, infection of humans with *T. suis* is usually self-limiting and infertile [16]. *T. trichiura* infects approximately 500 million people and is one of the most important soil-transmitted nematode infections [17]. Adult worms occupy the epithelial layer of the cecum and the proximal colon of the large intestine [17]. They are attached by the thinner anterior end.

1. Prostaglandin E2 (PGE2)

Extracted soluble products from the pig gastrointestinal nematode *T. suis* (TsSPs) were shown to suppress production of TNF-α and IL-12 from activated human dendritic cells [18]. Chromatographic separation and tandem mass spectrometry identified the active component as PGE2 [18]. TsSPs and human PGE2 both act through the EP2 and EP4 receptors on dendritic cells [18]. Host PGE2 promotes dendritic cell polarisation and favours a Th2 response although low doses promote Th1 and Th17 responses [18]. However, Th2 responses are thought to be protective in *T. suis* infections and the role of PGE2 may be to diminish pathology [18]. PGE2 is also released by the filarial nematodes *Brugia malayi* and *Wuchereria bancrofti* [19] but concentrations in fourth-stage larvae (L4) excretory/secretory (E/S) products from another pig intestinal nematode—*Ascaris suum* were very low [18]. These results suggest that some but not all parasitic nematodes use PGE2 to modulate dendritic cell activity.

2. Chitinase

Another immunomodulator is produced by the L1 of *T. suis* [20]. ES proteins from L1 are known to reduce clinical signs in a murine ovalbumin-induced allergic airway disease model (AAH). A comparison of ES proteins from L1, L2, L3 and L4 larvae identified proteins specific to early stage larvae [20]. Six proteins were cloned and expressed in a *Leishmania* expression system [20]. One protein was shown to reduce eosinophil infiltration into the mouse lung in the AAH model [20]. This protein is a chitinase and is similar in sequence and structure to helminth chitinases from *T. trichiura*, *A. suum* and *Necator americanus* [20]. The mechanism of immunomodulation is not known but it does not appear to require chitinase activity because heat inactivation did not abolish the immunomodulatory effects [20]. There was no detectable effect on activation of dendritic cells or T cell proliferation but there was an increase in IL-18 and IL-4 as well as Th2 cells producing IL-13 and expressing GATA-3 [20]. Antibodies to acidic mammalian chitinase (AMCase) decrease eosinophilia in AAH and AMCase interacts with the EGF receptor [20]. One intriguing possibility is that helminth chitinase interferes with EGFR binding by AMCase [20].

3. Triosephosphate isomerase (TPI) and Nucleoside diphosphate kinase (NDK)

*T. suis* also produces two proteins that modulate cytokine production [16]. Stimulation of human bone marrow-derived macrophages (BMDM) with interferon gamma (IFN-γ) and LPS resulted in the release of TNF-α but preincubation with excretory/secretory products from *T. suis* (TsESP) inhibited TNF-α release and increased the release of IL-10 [16]. Similarly, TsESP decreased IL-12p70 release following stimulation with CpG-ODN [16]. TsESP induced both Nitric oxide and arginase-1 expression suggesting that TsESP might induce myeloid-derived suppressor cells [16]. Fractionation of adult TsESP by gel permeation chromatography followed by proteomic analysis and the production of selected recombinant proteins implicated triosephosphate isomerase (TPI) and nucleoside diphosphate kinase (NDK) as the proteins responsible for immunomodulation. The enzyme activity of both proteins are known: TPI catalyses the isomerisation of dihydroxyacetone phosphate to glyceraldehyde-3 phosphate during glycolysis while NDK catalyses the transfer of phosphate and is required during nucleic acid synthesis [16]. However, it is not clear how these enzymes modulate the immune response. Treatment of BMDM with adult TsESP, TPI or NDK resulted in the phosphorylation of STAT3 which is a transcription mediator that drives the expression of immune response related genes [16].

4. Interferon gamma mimic

Many strains of mice mount a Th2 response following infection and expel *T. muris* while others mount a Th1 response and develop a chronic infection [21]. The Th1 response is dependent on the production of IFN-γ [21]. *T. muris* produces a molecule which cross-reacts with antibodies to murine IFN-γ, binds the IFN-γ receptor and produces similar effects on murine lymphoid cells to host IFN-γ [21].

5. The protein p43

IL-13 plays an important role in the development of the Th2 response and it is essential for the protective immune response in mice against *T. muris* [22].

A single protein dominates the excretory/secretory proteins of *T. muris* [17]. This protein is monomeric and has an approximate molecular weight of 43kDa by SDS PAGE [17]. It has 397 amino acids and 18 disulphide bonds; it is N-glycosylated at positions 57 and 287 [17]. X ray crystallography showed 16 β-strands and 6 α-helices [17]. The molecule binds both IL-13 and glycosaminoglycans and interferes with cytokine production by cells from infected mice [17]. These results are consistent with the molecule tethering IL-13 to the extracellular matrix and interfering with the priming of CD4+ helper cells [17]. Although p43 is immunogenic and vaccination with this molecule can protect against infection, it does not normally provoke an immune response against itself [17]. Sequence similarity searches showed similar molecules in other *Trichuris* species and in related *Trichinella* species [17].

A recent submission to bioRxiv suggested that p43 is a dorylipophorin [23]. These molecules have been found only in clade 1 nematodes and carry lipids. The orthologous molecule from *T. trichiura* (p47) was cloned and expressed. It too was a lipid binding protein. The lipids might contribute to the immunomodulatory activity of p43 [23].

6. Serine Protease Inhibitor (Serpin)

The genus Trichinella contains several species and genotypes [24]. They are intracellular parasites of skeletal muscle. Infection follows ingestion of animal tissue containing larvae [24]. *Trichinella pseudospiralis* is a cosmopolitan nematode whose larvae do not encapsulate after muscle cell differentiation. A serpin was cloned and characterised; the recombinant protein has 387 amino acids with a molecular mass of 43 kDa [24]. Modelling suggested the existence of α-helices and β-strands and a functional reactive site loop near the C terminus [24]. Western blotting of ES proteins suggested that the serpin was secreted by all developmental stages [24].

In vitro testing indicated that the recombinant molecule was able to inhibit the activity of porcine pancreatic elastase as well as human neutrophil elastase; the ability to inhibit digestive enzymes could prevent damage to *T. pseudospiralis* [24]. The recombinant serpin was also shown to inhibit mouse mast cell protease-1 (mMCP-1) but had little effect on human neutrophil cathepsin G [24]. Mast cell proteases break down tight junctions between epithelial cells which allows the egress of antibodies and other immunologically active molecules and cells [25,26]. The inhibition of mMCP-1 could reduce immune attack [24].

In vitro culture with murine J774A.1 macrophages showed that the recombinant serpin induced polarisation of macrophages to the M2 phenotype [24]. Western blotting revealed that increasing doses of recombinant serpin were accompanied by increased phosphorylation of Janus tyrosine kinase 2 (JAK2) and signal transducer and activator of the transcription 3 (STAT3) implying that the serpin activated the JAK2/STAT3 pathway [24] to influence production of cytokines and macrophage polarisation [24].

Clade III nematodes include the filarial nematodes. They are a major scourge of humans; the three most important filarial nematodes responsible for lymphatic filariasis are *W. bancrofti*, *B. malayi* and *Brugia timori.* Mosquitoes acquire microfilariae when they ingest blood from an infected individual. The microfilariae develop into infective larvae and are transmitted when an infected mosquito bites another human. The larvae migrate to the lymphatic vessels, mature into adult worms and produce microfilariae which circulate in the blood.

Subcutaneous filariasis includes onchocerciasis which is caused by *Onchocerca volvulus* and can cause river blindness. Filarial nematodes also infect domestic animals including cattle, sheep, dogs and rodents. *Acanthochielonema viteae* is used as a model species.

Other clade III nematodes include the large parasite of the porcine small intestine *A. suum* and a similar parasite of humans *Ascaris lumbricoides.* Another clade III nematode is *Toxocara canis* which infects the small intestine of dogs but can also infect humans and other animals.

7. Abundant larval transcripts 1 and 2 in *B. malayi*

*B. malayi* causes long-lived infections that are accompanied by changes in macrophage and T cell activity [27]. Although it is a human parasite, it can be used to infect mice. Approximately 5% of mRNA transcripts from infective larvae are from two related genes: abundant larval transcript-1 and -2 [27]. These two genes *Bmalt-1* and *Bmalt-2* code for proteins that show 79% amino acid identity [27]. Similar genes are present in other filarial nematodes and these proteins can generate protective immunity in animal models [27]. The two genes were expressed in the protozoan parasite *Leishmania mexicana* [27]. In vitro infection of bone marrow-derived macrophages was more successful with Bmalt-1 and Bmalt-2 transfected amastigotes; the transfected protozoa infected more cells and the infected cells had more parasites [27]. The transfected protozoa also produced larger lesions more quickly in infected mice [27]. Transfection did not alter production of nitric oxide by infected macrophages and did not alter expression of iNOS, IL-10 or IL-12. The transcription factor GATA-3 influences the Th2 response; expression drops in macrophages infected with wild type protozoa but is maintained in macrophages infected with *Bmalt-1* or *Bmalt-2* transfected protozoa [27]. Expression of the protein Suppressor of Cytokine Signalling-1 (SOCS-1) is also affected by infection. SOCS-1 is upregulated in macrophages 24 h after infection but expression remains elevated only in macrophages infected with transfected protozoa and not in macrophages infected with wild type protozoa [27]. Infection of mice with *B. malayi* confirmed that expression of both GATA-3 and SOCS-1 is increased following infection in both macrophage-enriched adherent cells and lymphocyte enriched non-adherent cells [27].

8. Cystatins

Cysteine proteases are involved in a wide variety of physiological procedures including antigen processing [28]. Their inhibitors are called cystatins and they play a crucial role in regulating proteolysis [28]. Nematodes also produce cystatins and they influence Class II MHC restricted antigen processing [29]. Two genes (Bm CPI-1 and Bm CPI-2) with sequence similarity to mammalian cystatins were isolated from *B. malayi* [29]. Recombinant Bm CPI-2 was able to inhibit proteolysis of three fluorogenic substrates that were preferentially cleaved by the cysteine proteases cathepsin S, cathepsin B/L and asparaginyl endopeptidase [29]. Bm CPI-2 was also able to inhibit processing of the tetanus toxin C fragment antigen in vitro [29]. Additional experiments with EBV-transformed B cells showed that Bm CPI-2 was able to inhibit antigen presentation to T cells [29]. CPI-2 is one of the most abundant transcripts made by *B. malayi* larvae [29] and as filarial larvae reside in lymph, CPI-2 is likely to be taken up by antigen presenting cells in lymph nodes [29].

Immunomodulatory cystatins have been described in a variety of other nematodes including *A. viteae* [30], *O. volvulus* [31], *Litomosoides sigmodontis* [32], *Heligmosomoides polygyrus* [33], *Nippostrongylus brasiliensis* [34], and *A. lumbricoides* [35,36,37]. These results showed that the effect of nematode derived cystatin was not restricted to antigen processing; they also influenced production of IL-10 and TNF-α as well as T cell proliferation. Cystatin is made by filarial nematodes and *A. lumbricoides* from clade III, as well as *H. polygyrus* and *N. brasiliensis* from clade V. Therefore, cystatin is probably produced by a wide variety of nematodes and may modulate the immune response in a variety of infections.

9. Tgh-2

Screening the EST database generated by the filarial genome project identified a gene with a similar amino acid sequence to human TGF-β2; 37% amino acid similarity in the C-terminal ligand domain [38]. The full length DNA sequence was expressed in a baculovirus and the recombinant protein showed a low level of binding to mink lung epithelial cells expressing the TGF-β receptor [38]. This binding was partially inhibited by human TGF-β [38]. As TGF-β influences the immune response [39], these results suggest that Tgh-2 may modulate the immune response.

10. Asparaginyl t-RNA synthetase

Filarial nematodes modulate immune responses especially CD4+ T cells and IL-10 responses [40]. Following infection, some individuals develop lymph vessel hyperplasia and lymphangiogenesis which underlie elephantiasis; NF-κβ and IL-8 mediated production of vascular endothelial growth factor (VEGF) has been implicated [40]. Asparaginyl t-RNA synthetase binds IL-8 receptors and recombinant Asparaginyl t-RNA synthetase from *B. malayi* (rBmAsnRS) was used to induce recovery from a murine T cell transfer model of colitis [40]. In addition, rBmAsnRS stimulated multiple signal transduction pathways in immature human dendritic cells including Toll-like receptor, MAPK and NK cell mediated signalling [40]. rBmAsnRS also upregulated IL-10 and Il-22 receptors [40].

11. ES-62

ES-62 is an excretory–secretory molecule of the filarial nematode *A. viteae*. Its molecular weight is approximately 62kDa [41]. It has multiple Phosphorylcholine (PC) moieties attached to N-type glycans [42]. It suppresses inflammation in mouse models of asthma and collagen induced arthritis [42,43] possibly by reducing Th17-mediated inflammation and by resetting the Th1/Th2 balance. It can act via TLR-4 and MyD88 signalling [44]. It affects multiple immune cells; it reduces the ability of dendritic cells to prime naïve CD4+ cells, it reduces IL-17 production by Th17 cells, it reduces mast cell activity and it suppresses the recruitment of eosinophils and neutrophils to the lung in mouse models of asthma [42].

ES-62 also affects signalling between IL-33 and its receptor [45]. IL-33 and its receptor, like TLR-4, uses MyD88 to relay signals to intracellular molecules. Therefore, there are potential synergies between these pathways [45]. ES-62 uses different mechanisms to inhibit LPS/TLR-4 dependent cytokine responses in serosal derived mast cells and bone-marrow derived mucosal mast cells [45]. Administration of ES-62 or genetic deficiency of the IL-33 receptor was associated with reduced levels of allergen-specific IgE in a chronic Ovalbumin/alum mouse model of asthma [45].

12. Nematode-derived Migration inhibitory factor (nMIF)

Macrophages are a heterogeneous collection of tissue resident cells that play important roles in host defence. In the Th1 environment commonly found in resistant individuals following bacterial infection, macrophages are activated into M1 cells; they present antigen and phagocytose bacteria. In the Th2 environment that occurs in relatively resistant hosts following nematode infection, macrophages develop into M2 cells. These cells can promote peristalsis and facilitate the expulsion of adult nematodes and assist in the killing of tissue dwelling larvae, at least in rodent models [46]. M2 macrophages also participate in tissue repair [47]. Both M1 and M2 cells regulate the activity of innate and adaptive immune cells [11].

Macrophage migration inhibitory factor (MIF) is a pleiotropic cytokine that regulates macrophages and influences the immune response. MIF assists in cell recruitment to the site of infection, it can promote the polarisation of macrophages to M2 cells, it inhibits the suppression of immune responses by glucocorticoids and influences the release of immune mediators by macrophages. MIF is required for protective immune responses against the nematode *Heligmosomoides polygyrus* [48] but MIF deficient mice have more effective responses against the gastrointestinal rodent nematode *N. brasiliensis* [49].

MIF is a homotrimer that binds CD74 (MHC II invariant chain) complexes with CD44 and phosphorylates ERK1/2 (proline-directed kinases) [50]. The receptor complex can also be associated to the chemokines CXCR2 and CXRC4 [50].

Most of the nematodes that have been studied possess at least two MIF-like genes: MIF-1 and MIF-2 [11] with low amino acid sequence (20–42%) similarity to mammalian MIF although MIF-1 is more similar than MIF-2 [11]. Despite the low sequence similarity, mammalian and nematode MIF are structurally similar and MIF from several nematode species binds to human CD74 [11].

Nematode MIF, like mammalian MIF, has multiple functions and many of these functions are similar although there are some differences among different nematode species. Mammalian MIF is a tautomerase and an oxidoreductase and nematode MIF can have similar but weaker enzyme activity [11]. Recombinant MIF from *B. malayi*, can, like human MIF, promote the chemotactic migration of distant macrophages and inhibit the random movement of nearby macrophages [11,51]. Recombinant MIF from the nematode *Trichinella spiralis* influences the migration of human peripheral blood mononuclear cells, PBMC [11]. Both mammalian and nematode MIF can bind the human transcription factor Jun activation domain-binding protein 1 (JAB1) [11]; JAB1 influences the expression of genes that affect apoptosis and inflammation. Recombinant MIF from *B. malayi* can also promote the release of MIF from human monocytes, promote polarisation of M2 macrophages and recruit eosinophils [11]. Both nematode and host MIF stimulate the release of various cytokines including TNF-α and IL-8 [11].

PAS-1

Protein 1 from *A. suum* (PAS-1) is an ES protein produced by larvae and adults [52]. Purified PAS-1 suppresses the humoral immune response to ovalbumin and the inflammatory response to LPS in a mouse model [52]. The mechanism appears to be IL-10 dependent and may involve regulatory T cells [52]. The 11 N terminal amino acids were sequenced by Edman degradation and were identical to the previously described ABA-1 polyprotein [53].

Mucins

Mucins are highly glycosylated proteins that have multiple functions including lubrication of the intestines. Mucins-2, -3, -4 and -5 from the intestinal nematode *T. canis* were cloned and expressed in yeast [54]. Spleen cells from *T. canis* infected mice express IL-4, IL-5 and IL-10 and production is increased by ES products or by recombinant mucins [54]. Cells from infected animals produced less IFN-γ and IL-17 suggesting that the nematode mucins influence the upregulation of Th2 responses and the downregulation of Th1 and Th17 responses.

The clade V nematodes include hookworms from the superfamily Ancylostomatoidea which includes the dog hookworm *Ancylostoma caninum*, the zoonotic hookworm *A. ceylanicum* and the human hookworms *A. duodenale* and *N. americanus*. [14]. Hookworm heads are shaped like hooks and the adult worms live in the small intestine. Hookworms infect approximately 400–500 million people [55]. They are spread by walking barefoot on or by ingesting contaminated soil. The worms feed on blood, and heavy infections can cause severe anaemia and protein deficiency.

Other nematodes in clade V include *H. polygyrus* which is a natural parasite of wood mice and is widely used as a model species as well as another model species *N. brasiliensis.* Clade V also includes the economically important parasites of livestock *Haemonchus contortus* and *Teladorsagia circumcincta*. *H. contortus* and *T. circumcincta* seldom occur together; *H. contortus* is found in hotter climates such as Kenya, Australia and Mediterranean countries, while *T. circumcincta* occurs in cooler areas such as Southern Australia and Northern Britain. Both *H. contortus* and *T. circumcincta* can cause relative protein deficiency in growing sheep and this is exacerbated by blood loss with *H. contortus.*

Astacin

Astacins are a family of zinc metalloproteases [56] and at least four have been described in the genus Ancylostoma: Ace-MTP-1 and Ace-MTP-2 from *A. ceylanicum* and Ac-MTP-1 and Ac-MTP-2 from *A. caninum* [57]. AceMTP-1 and AcMTP-1 are similar as are AceMTP-2 and AcMTP-2 [57]. AceMTP-2 has been cloned, sequenced and expressed in *Escherichia coli* and *Pichia pastoris*; it has a 16 amino acid signal peptide followed by a 214 aa protein with a calculated mass of 24.96 kDa that contains a zinc-dependent metalloprotease domain [57]. The gene is predominantly expressed in adult parasites and secreted [57]. The recombinant protein amplified the in vitro release of TNF-α and induced the release of IFN-γ by LPS-activated THP-1 macrophages [57].

Peptides that block the voltage-gated potassium channel 1.3 (Kv1.3)

The voltage-gated potassium channel Kv1.3 regulates the membrane potential of T cells by allowing potassium ions to leave the cell to balance the incoming calcium ions during signalling [58]. Molecules that inhibit Kv1.3 suppress proliferation and cytokine production by effector memory T cells (Tem) and reduce clinical signs in rodent models of autoimmune diseases [58]. One inhibitor is SHK and the SMART tool predicts the existence of over 600 proteins with domains resembling SHK including nearly 300 from helminths [58]. The anterior secretory glands from *A. caninum* were used to create a cDNA library; over 2000 clones were sequenced and revealed 159 unique transcripts [58]. The sequence of an abundant transcript AcK1 was used to search genomic databases and 53 similar proteins were identified from a variety of helminths [58]. *A. ceylanicum* contained an identical molecule (AceK1) [58]. *B. malayi* contained a similar domain in a zinc metalloprotease (BmK1) [58]. Both molecules were synthesised along with a molecule (BmK2) differing by five amino acids to maximise the interaction with Kv1.3 and their structures determined [58]. AcK1 and BmK2 blocked Kv1.3 and suppressed proliferation of a rat Tem cell line. Bmk2 also suppressed delayed type hypersensitivity reactions in rats [58]. Phylogenetic analysis identified similar molecules to AcK1 in related species of nematodes; the molecule from the related nematode *T. circumcincta* was synthesised and it suppressed IFN-γ production but had no effect on IL-4 or IL-17A production [59].

Tissue inhibitor of metalloprotease-1 and -2

Proteomic analysis of ES from *A. caninum* identified two abundant proteins: Tissue Inhibitor of metalloprotease-1 and Tissue Inhibitor of metalloprotease-2 [60]. These proteins are also known as AIp-1 and AIp-2 for anti-inflammatory proteins [60].

Pre-treatment with recombinant TIMP-1 of mice with experimental colitis reduced the mucosal pathology [60] while splenocytes from pretreated mice produced less TNF-α and more IL-10 than untreated mice following stimulation with anti-CD3 [60]. The native protein contains an asparagine at position 119 which appears to be glycosylated by *Pichia* in the recombinant protein; to remove the effect of glycans, a modified protein was created with glutamine substituted for asparagine [60]. This modified protein suppressed the production of IL-13, IL-17A and IFN-γ in diseased colons but increased TSLP suggesting increased healing [60]. Naïve mice treated with the modified recombinant protein also had increased numbers of CD4^+^CD25^+^FoxP3^+^ cells [60] suggesting that AIP-1 increases the number of regulatory T cells [60].

Recombinant TIMP-2 induced the expansion of CD11c+ CD103+ dendritic cells in mice which generate Treg [61]. It also decreased the expression of MHC class II molecules on DC [61]. TIMP-2 suppressed the infiltration of eosinophils and lymphocytes and suppressed airway inflammation in a mouse model of asthma [61]. Both TIMP-1 and TIMP-2 appear to induce regulatory T cells raising the possibility that the two molecules may act synergistically to suppress immune responses [60].

Neutrophil Inhibitory Factor (NIF)

A 41Kda glycoprotein was isolated and cloned from *A. ceylanicum* [62]. This molecule had 274 amino acids with a calculated mass of 28,926 daltons and seven predicted N-glycosylation sites [62]. It bound the CD11b/CD18 complex on neutrophils and monocytes and inhibited two different CD11b/CD18 mediated functions: adhesion of activated neutrophils to endothelium and adherence dependent release of hydrogen peroxide from stimulated neutrophils [62].

A similar molecule was isolated from ES products of the related gastrointestinal nematode *H. contortus* [63]. This molecule had an apparent molecular weight of 55 kDa but cross-reacted with antibodies against NIF from *A. ceylanicum*, it bound CD11b/CD18 and inhibited the release of hydrogen peroxide from activated neutrophils [63]. This molecule also inhibited the release of hydrogen peroxide from monocytes but the effect was more noticeable in unstimulated cells compared to PMA-activated monocytes [63].

Metalloprotease

Eotaxin and LTB_4_ stimulate the recruitment of eosinophils to the site of injection in guinea pigs [64]. This eosinophil recruitment by eotaxin is diminished by pre-incubation with ES products from the adult hookworm *N. americanus* although the ES products had no effect on eosinophil recruitment by LTB_4_ [64]. The ES products caused a loss of eotaxin reactivity in an ELISA but there was no effect on eotaxin-2 or IL-8 [64]. Gel filtration chromatography showed that the activity existed in two sets of fractions; one with an approximate molecular weight of 15kDa and one with a molecular weight of approximately 50 kDa [64]. This could imply two distinct molecules or post translational modification such as glycosylation or the formation of multimers [64]. The inhibition of eotaxin was prevented by EDTA but not EGTA implying that the inhibition was not Calcium dependent; the inhibition was blocked by phenanthroline, implying that the molecule (or molecules) was a metalloprotease [64].

Activation-associated protein 2 (ASP-2)

Two groups have looked at Na-ASP-2 from *N. americanus*. One group created air pouches in mice; injection of Na ASP-2 caused a transient infiltration of leukocytes which were 60% neutrophils [65]. An in vitro study using Boyden chambers showed that ASP-2 recruited only neutrophils [65]. Larvae that migrate through tissues often cause inflammation, possibly to increase tissue permeability and permit faster migration of larvae [65]. Neutrophil recruitment could be part of this process.

Another group probed a human proteome array with biotinylated Na-ASP-2 and showed binding to CD79A which is a subunit of the heterodimeric B cell antigen receptor [65,66]. CD79A is expressed only on B lymphocytes and Na Asp-2 binds selectively to B lymphocytes and suppresses the expression of multiple genes including the B cell receptor signalling pathway [66]. Neutrophils compete with APC for antigen; therefore, neutrophil recruitment could suppress B and T cell responses [66].

Alarmin release inhibitor (HpARI)

Following gastrointestinal infection, epithelial cell injury results in the release of alarmins [67]. Three alarmins initiate Th2 immune responses: thymic stromal lymphopoietin (TSLP), IL-25 and IL-33. IL-33 is particularly important for resistance to nematode infection. Mice lacking IL-33 expel the small intestinal nematode *H. polygyrus* more slowly. In addition, giving exogenous IL-33 to mice infected with this parasite promotes parasite expulsion [68]. IL-33 has been recently reviewed [67]. IL-33 has 270 AA in humans and 266 AA in mice [67]. IL-33 is released by epithelial and endothelial cells as well as mast cells [67,69]. Its receptor is expressed on epithelial and endothelial cells as well as group 2 innate lymphoid cells (ILC2), T cells and macrophages [67]. IL-33 is stored in the nucleus [70]. Active IL-33 is released as a reduced molecule that is rapidly oxidised [68]. Consequently, it is active in a limited area and for a limited time after release. Regulatory T cells can also bind IL-33; therefore, this molecule can increase or decrease resistance to nematode infection [71].

*H. polygyrus* produces HpARI which binds both active IL-33 and murine DNA effectively tethering IL-33 to the cell nucleus in necrotic cells and inhibiting the development of Th2 responses [68,72].

Three HpARI family members were identified by searching genomic and transcriptomic data [71]. All contain a signal peptide and three complement control protein domains [71]. The three proteins contain 248 to 251 amino acids and their sequence identity varies from 69 to 81% [71]. They all bind IL-33; HpARI1 and HpARI2 suppress ILC2 responses and eosinophilia while HpARI3 enhances ILC2 activity and eosinophilia following exposure to *Alternaria* allergen [71]. HpARI3 binding stabilises IL-33 and extends its active life [71].

*H. polygyrus* Binds Alarmin receptor and Inhibits (HpBARI)

*H. polygyrus* also secretes a molecule that binds to the IL-33 receptor complex (ST2 and IL-1 receptor accessory protein) and inhibits Il-33 mediated responses [73]. This molecule also contains, like HpARI, two atypical CCP domains [73]. A codon-optimised sequence was cloned and expressed in mammalian cells. It has a predicted molecular weight of 23KDa and migrated at approximately 30kDa on SDS gels [73]. The purified recombinant molecule suppressed expression of the IL-33 receptor on ILC2 cells and inhibited the production of IL-5, IL-6 and IL-13 in vitro by naïve bone marrow cells treated with IL-2, IL-7, and IL-33. The recombinant HpBARI also suppressed bronchoalveolar lavage (BAL) and lung eosinophilia in an in vivo model when co-administered with *Alternaria* allergen. Searching the Wormbase Parasite revealed two molecules with BARI activity [73] with 58% amino acid identity. HpBARI can bind both membrane bound and soluble forms of ST2.

Small RNA molecules that suppress immunity

In addition to the two proteins that affect the IL-33 molecule and its receptor, *H. polygyrus* also expresses small RNA molecules that are secreted in exosomes [74,75]. In vitro experiments showed that the vesicles are taken up by intestinal epithelial cells and suppress expression of the IL-33 receptor [74]. The vesicles also suppress expression of *DUSP1* which regulates mitogen activated protein kinase (MAPK) pathway signalling [74]. Intranasal administration of exosomes suppresses the Th2 innate responses and eosinophilia induced by *Alternaria* allergen [74]. Therefore, *H. polygyrus* produces at least three sets of molecules that influence IL-33 signalling and the Th2 immune response.

TGF-β mimic (TGM)

Mice infected with *H. polygyrus* have increased numbers of Treg; depletion of Treg by specific antibody leads to worm expulsion while expansion of Treg makes resistant mice susceptible [76]. Transforming growth factor beta (TGF-β) induces Treg. ES products from *H. polygyrus* can also induce Treg [76]. Gel filtration and anion exchange FPLC were used to fractionate the approximately 400 proteins in the ES [76]. The fractions were then tested for their ability to activate the MFB-F11 fibroblast cell line [76]. The four proteins with the closest match between abundance and biological activity were then cloned; one protein was shown to signal through the TGF-β pathway [76]. This protein was named TGM (transforming growth factor mimic) and shown to induce Treg [76].

Subsequent research has identified 10 members of the TGM family [77]. TGM1 has 422 amino acids including a signal peptide of 18 amino acids [76]. It contains five CCP-like domains [76]. Although it is not similar in sequence to TGF-β, it binds directly to the TGF-β receptor complex [78]; domains 1 and 2 bind TGFBR1 while domain 3 binds TGFBR2. Domains 4 and 5 bind the co-receptor CD44 [77]. In other members of the TGM family, domains 4 and 5 bind coreceptors on other cells and determine which cells are affected by TGM [77]. TGM6, TGM9 and TGM10 lack domains 1 and 2 and cannot bind TGFBR1 but TGM6 can bind TGFBR2 and blocks TGF-β signalling in fibroblasts and epithelial cells [77]. The members of the TGM family may have evolved to initiate or prevent TGF-β signalling in different cell types [77].

Glutamate dehydrogenase (p66)

Anion exchange chromatography of adult *H. contortus* ES products followed by Con A sepharose and anion exchange chromatography isolated a 66 kDa protein [79]. This protein reacted with antisera from infected but not from uninfected goats [79]. The molecule enhanced the mitogen induced proliferation of peripheral blood mononuclear cells (PBMC), caused the proliferation of lymphocytes from uninfected goats and decreased the production of peroxide and nitric oxide by monocytes from uninfected goats [79]. Subsequently, the protein was identified as Glutamate dehydrogenase by mass spectrometry and cloned [80]. Both the native and the recombinant molecule stimulated proliferation of goat PBMC and the release of IL-4 and IFN-γ [80].

Acetylcholinesterase from *N. brasiliensis*

Following nematode infection, Tuft cells in the intestinal epithelium secrete IL-25; this activates ILC2 which secrete IL-4 and IL-13. This triggers the Th2 response [81]. Tuft cells also release acetylcholine which inhibits egg laying by acting on muscarinic receptors on the nematode [82]. Many species of nematode secrete acetylcholinesterases [83]. Acetylcholinesterase from *N. brasiliensis* was expressed in the natural mouse parasite *Trypanosoma musculi* [83]. Splenocytes from infected mice expressed less IL-4 and IL-13 but more IFN-γ and TNF-α [83]. M1 macrophages were activated with enhanced production of nitric oxide and decreased production of arginase [83]. Mice infected with transgenic *T. musculi* cleared the nematode infection quicker [83].

Apyrases

Extracellular ATP is important. In Spi-B deficient mice, following deliberate infection with *H. polygyrus*, fewer worms established and fewer eggs were produced [69]. The Spi-B deficient mice had goblet cell hyperplasia and rapidly expressed *IL-13* but not *IL-4* [69]. There was an increase in ILC2 cells and significantly more mast cells [69]. Elimination of mast cells with anti-c-Kit antibody removed the relative resistance of the Spi-B deficient mice and depleted the ILC2 cells [69]. Spi-B deficient mice contained more mast cells expressing IL-33 which is required for ILC2 activation [69]. Addition of ATP to cultured mast cells led to activation and degranulation [69]. ATP could come from epithelial cells damaged during *H. polygyrus* infection [69]. Apyrases digest ATP and could inhibit the release of IL-33 by mast cells. Many nematodes including *H. polygyrus* possess apyrases [84].

Galectin

Galectins bind beta-galactosides and influence many cellular functions. Some members of the family bind intracellular glycans while other bind extracellular glycans [12]. They are present in nearly all animals including mammals and nematodes [12]. They bind a wide variety of molecules including antibodies and mucins and play important roles in defence against nematode infection [12]. Among the more critical effects of nematode galectin are inhibition of mast cell degranulation [85], the induction of apoptosis [86,87] and the thickening of gastrointestinal mucus [12].

Both hosts and nematode produce multiple distinct galectin molecules [12] and they have a complex evolutionary relationship [88]. Although their sequence similarity is low, their carbohydrate recognition domains are similar in structure [12]. The amino acids in human galectin-3 that bind carbohydrate have been identified and sequence alignment indicates that the corresponding amino acids are identical in galectin-1 from the sheep nematode *T. circumcincta* [12,85].

**Figure 1:**
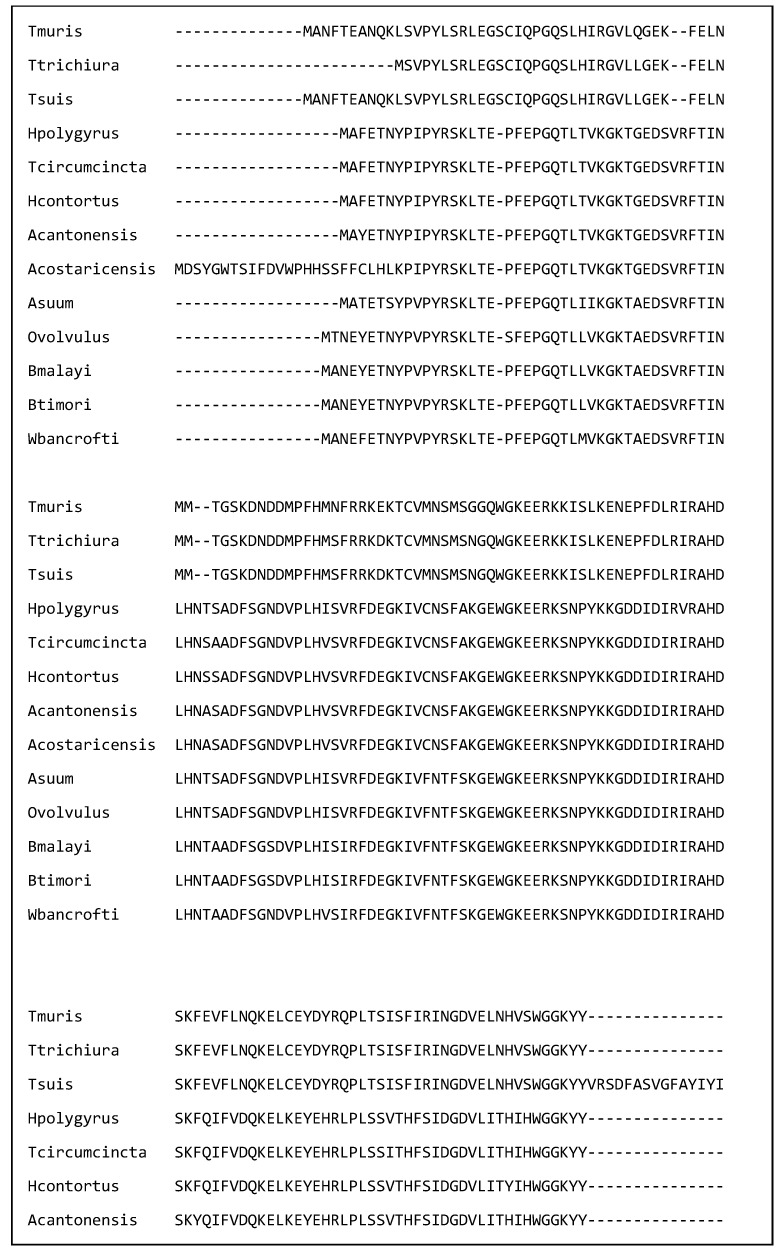

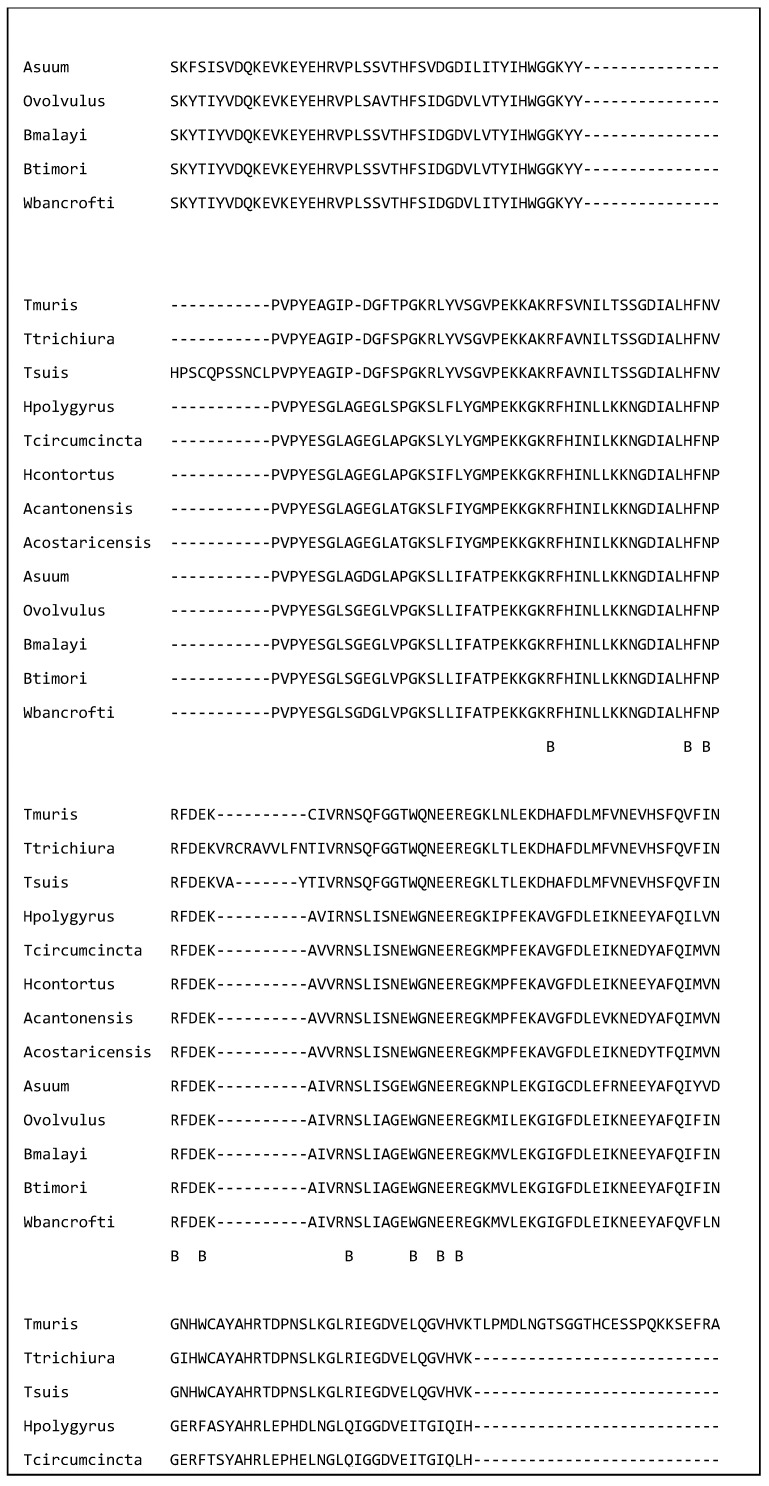

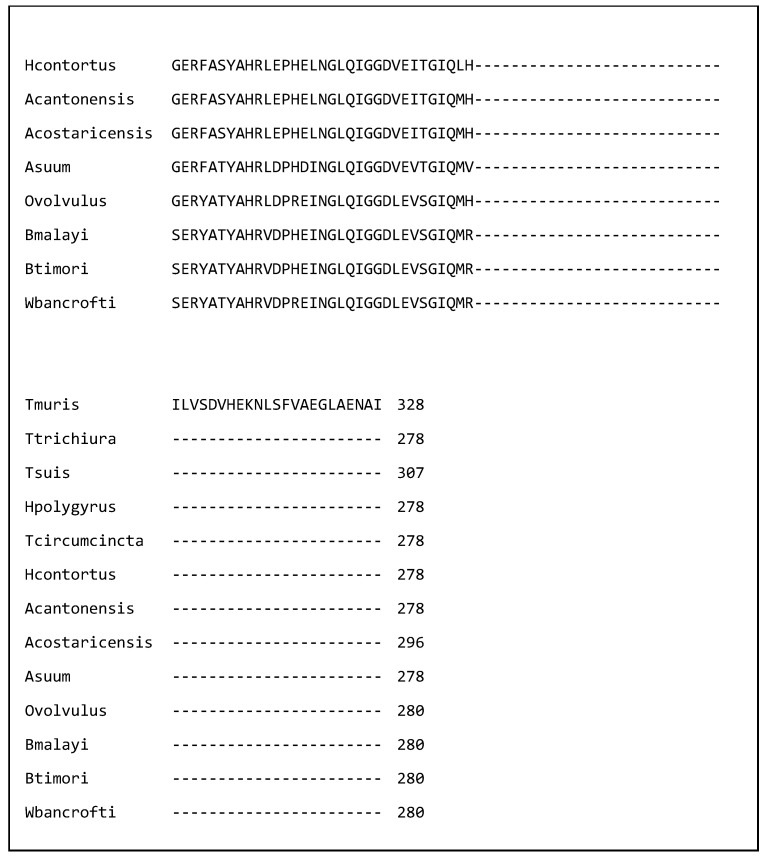
A multiple sequence alignment of 13 nematode galectins showing that the amino acids in the putative binding site (labelled ‘B’) are identical. The alignment was created in Clustal omega on the European Bioinformatics Institute webpage on the 2 March 2025. The sequences are *T. muris* (A0A5S6Q6D7), *T. trichiura* (A0A077YZM7), *T. suis* (A0A085MAM6), *H. polygyrus* (A0A3P8DGW1), *T. circumcincta* (O01411), *H. contortus* (UPI000007AE4F), *A. cantonensis* (UPI0007A21A4B), *A. costaricensis* (A0A3P7H3L8), *A. suum* (F1KZZ8), *O. volvulus* (Q25597) *B. malayi* (A0A0H5S1P8), *B. timori* (A0A0R3QLT7), and *W. bancrofti* (A0AAF5PVA7).

Figure 1 shows a multiple sequence alignment of galectins from 13 of the nematode species discussed in this review. The amino acids in the putative carbohydrate binding site have been indicated by a B. Each amino acid is identical in all 13 species. This identity suggests that they could bind similar glycan residues. Further the amino acid identity supports the hypothesis that galectins from a variety of species have similar immunomodulatory effects and galectins from a variety of nematode species could be used to treat immune disorders.

## 3. Discussion

The discovery of immunomodulatory molecules in nematodes is not too surprising because a wide variety of pathogenic organisms including viruses, bacteria and flatworms have been shown to suppress the immune response [89,90,91]. What is surprising is the way nematodes suppress the immune response. The surprises can be grouped under three headings: complexity, diversity and mechanistic synergy.

Immunomodulation by nematodes is surprisingly complex. The most studied nematodes in the area of immunomodulation are *H. polygyrus*, *B. malayi* and the *Trichuris* spp. [1]. At least six immunomodulatory molecules have been identified for these taxa and it is likely that additional molecules exist. This is good news for those looking for ways to suppress the immune response in patients suffering from undesirable immune responses. The production of multiple molecules by each species does raise problems though. A naïve view might expect only a limited number of molecules to be produced because each molecule incurs an energetic cost; evolution generally favours efficiency and works to eliminate redundant molecules. We do not know why nematodes produce multiple immunomodulatory molecules. Possible explanations include reduced energetic cost for parasites which obtain food from their hosts or the need to overcome immune responses from the host against individual molecules. As the immune system can respond to multiple molecules simultaneously, one strategy might be to produce immunomodulatory molecules sequentially. Another explanation for multiple immunomodulatory molecules is that they facilitate more subtle immune regulation. As nematode infections are long-lived, completely shutting down the immune system might render hosts more susceptible to additional infections from the same or different species.

Different nematode species employ different strategies to modulate the immune response. Some immunomodulatory molecules are present in multiple species including Macrophage migration inhibitory factor [11] and galectin [12]. Others have, so far, only been reported to be active in one or a few species [68]. Again, this is surprising. A simplistic approach might expect nematodes to evolve a limited number of effective mechanisms shortly after becoming parasites and to keep these as they speciate. There are several possible explanations. It may be that different nematode species provoke different immune responses which then require different sets of immunomodulators. For example, macrophages reside in the tissue and can kill larvae from those nematodes whose larvae migrate through tissue such as *H. polygyrus*, *N. brasiliensis* and *L. sigmodontis* [46]. In contrast, macrophages do not appear to be critical in *T. muris* infection [92]. Suppressing macrophage activity could be important for some but not all parasitic nematode species. Another explanation is that fully developed immune responses have evolved relatively recently and some immunomodulatory molecules may have evolved after the evolution of the major nematode clades and families.

The third major surprise is the mechanistic synergy of nematode-derived immunomodulators. For example, *H. polygyrus* produces a cysteine protease inhibitor that affects the differentiation of bone-marrow derived dendritic cells [33], an alarmin release inhibitor that prevents the release of IL-33 from epithelial and other cells [68], an miRNA that reduces expression of the Il-33 receptor [74], galectin that decreases mast cell degranulation [1] and a mimic of TGF-β that influences the differentiation and activation of regulatory T cells [78]. These immunomodulatory activities synergise. Regulatory T cells have receptors for IL-33 and IL-33 induces Tregs [67]. Mast cells are activated by IL-33 and influence its activity by the release of proteases [67]. An unsophisticated expectation is that nematodes would use multiple molecules to shut down different pathways of the immune response. Clearly, this is not happening. Possibly, the components of the immune system are too interconnected to target separate pathways. The assumption that the immune system can be decomposed into distinct pathways could be wrong. Alternatively, mechanistic synergy may provide more effective control over key elements of protective immunity.

The discovery of immunomodulators produced by nematodes is relatively recent and there are a number of caveats. Firstly, the evolution of most immunomodulators is unknown. Some potential immunomodulators are only present in a few species [93] and are assumed to have evolved relatively recently. Other molecules such as MIF are present in most nematodes [11] and are assumed to have evolved with the phylum Nematoda. However, the existence of a molecule with sequence similarity does not necessarily imply immunomodulatory activity and more functional studies are necessary. A better understanding of the evolution of immunomodulators will aid understanding of how they can be utilised to control aberrant immune activity or even help the development of urgently needed novel methods of parasite control.

There is considerable optimism that immunomodulatory molecules can be used to control the immune response and there have been multiple trials with nematode-derived molecules. However, nematodes do not rely on just one molecule to modulate the immune response and perhaps trials with multiple molecules could achieve better results. In addition, further research into the molecular mechanisms of action could lead to the production of novel, synthetic molecules that combine the most desirable features of orthologous molecules from several species [60].

Many of the immunomodulators act by influencing T cells, macrophages and dendritic cells. Only a small number of molecules influence B cells and the production of antibodies although antibodies, particularly IgA and IgE, play an important role in resistance to nematode infection [94]. This could be because dendritic cells, macrophages and T cells have key roles in regulating immune responses or because antibody responses are needed to fight potential concurrent infections by other pathogens. However, the cells that have been studied may not be fully representative of the immune response and it is premature to conclude that nematode-derived molecules target only some immune cells.

Much of the excitement generated by the discovery of immunomodulators is due to their potential role in the control of human diseases caused by nematode infection or aberrant immune responses. In addition, nematode infections are a major threat to the livestock industries and food production [5], and immunomodulators could assist in the control of livestock infections. Molecules that inhibit the action of selected immunomodulators could reduce the establishment and survival of nematodes; this could be by the development of novel drugs or by designing multivalent vaccines that include immunomodulatory molecules [95]. In addition, much of the pathology in livestock is due to the immune response; activated mast cells release proteases that break down tight cell junctions between epithelial cells in the gastrointestinal tract [96]. This leads to a relative protein deficiency and reduced growth rates in young animals. Immunomodulators could reduce the immunopathology.

Even though many nematode immunomodulators remain to be discovered and their mechanisms unravelled, they are behaving in unexpected and interesting ways. More research is likely to lead to the discovery of new immunomodulators with novel mechanisms of action. In addition, more research on the mechanisms by which immunomodulators influence the immune system and how they interact with each other could produce more precise and specific protocols to alleviate immune mediated disorders. This is an exciting and important area of research that is likely to produce more insight and more surprises.

## Figures and Tables

**Table 1:** Nematode species producing immunomodulators and their targets.

Species	Name of Nematode Molecule	Target Cell or Molecule in the Host
*Trichuris* spp.; *Brugia malayi*; *Wuchereria bancrofti*.	Prostaglandin E2 (PGE2)	Dendritic cells
*Trichuris suis*	Chitinase	Eosinophils; Th2 cells.
*Trichuris suis*	Triosephosphate isomerase Nucleoside diphosphate kinase	Cytokine release
*Trichuris muris*	Interferon gamma mimic	Interferon gamma
*Trichuris muris*	P43	IL-13
*Trichinella pseudospiralis*	Serine protease inhibitor (Serpin)	Serine proteases
*Brugia malayi*	Abundant larval transcripts 1 and 2 (Bmalt-1; Bmalt-2)	Expression of GATA-3 and SOCS-1
*Brugia malayi*	Tgh-2	TGF-β
*Brugia malayi*	Asparaginyl t-RNA synthetase	Various
*Acanthocheilonema viteae*	ES-62	TLR4
Multiple species	Nematode-derived Migration inhibitory factor (nMIF)	Macrophages
*Ascaris suum*	Protein 1 from *A. suum* (PAS-1)	Regulatory T cells?
*Toxocara canis*	Mucins	Helper T cells
*Ancylostoma ceylanicum*	Astacin (AceMTP-2)	Macrophages
*Ancylostoma caninum; Brugia malayi*	Peptides that block the voltage-gated potassium channel 1.3 (Kv1.3)	T cells
*Ancylostoma caninum*	Tissue inhibitor of metalloprotease-1 and -2	Dendritic cells
*Ancylostoma ceylanicum*	Neutrophil Inhibitory Factor (NIF)	Neutrophils
*Necator americanus*	Metalloprotease	Eosinophils
*Necator americanus*	Activation associated protein 2 (ASP-2)	Neutrophils; B cells
Multiple species	Cystatins	Antigen presenting cells
*Heligmosomoides polygyrus*	Alarmin release inhibitor (HpARI)	IL-33
*Heligmosomoides polygyrus*	Binds Alarmin receptor and Inhibits (HpBARI)	IL-33 receptor
*Heligmosomoides polygyrus*	Small RNA molecules	Expression of IL-33 receptor; MAPK signalling
*Heligmosomoides polygyrus*	TGF-β mimic (TGM)	TGF-β
*Haemonchus contortus*	Glutamate dehydrogenase (p66)	Lymphocytes and monocytes
*Nippostongylus brasiliensis*	Acetylcholinesterase	Macrophages
Multiple	Apyrases	Mast cells
Multiple	Galectin	Mast cells, mucus
